# Postural control of a musculoskeletal model against multidirectional support surface translations

**DOI:** 10.1371/journal.pone.0212613

**Published:** 2019-03-06

**Authors:** Kohei Kaminishi, Ping Jiang, Ryosuke Chiba, Kaoru Takakusaki, Jun Ota

**Affiliations:** 1 Department of Precision Engineering, School of Engineering, The University of Tokyo, Tokyo, Japan; 2 Research into Artifacts, Center for Engineering (RACE), The University of Tokyo, Kashiwa, Japan; 3 Research Center for Brain Function and Medical Engineering, Asahikawa Medical University, Asahikawa, Japan; Toronto Rehabilitation Institute - UHN, CANADA

## Abstract

The human body is a complex system driven by hundreds of muscles, and its control mechanisms are not sufficiently understood. To understand the mechanisms of human postural control, neural controller models have been proposed by different research groups, including our feed-forward and feedback control model. However, these models have been evaluated under forward and backward perturbations, at most. Because a human body experiences perturbations from many different directions in daily life, neural controller models should be evaluated in response to multidirectional perturbations, including in the forward/backward, lateral, and diagonal directions. The objective of this study was to investigate the validity of an NC model with FF and FB control under multidirectional perturbations. We developed a musculoskeletal model with 70 muscles and 15 degrees of freedom of joints, positioned it in a standing posture by using the neural controller model, and translated its support surface in multiple directions as perturbations. We successfully determined the parameters of the neural controller model required to maintain the stance of the musculoskeletal model for each perturbation direction. The trends in muscle response magnitudes and the magnitude of passive ankle stiffness were consistent with the results of experimental studies. We conclude that the neural controller model can adapt to multidirectional perturbations by generating suitable muscle activations. We anticipate that the neural controller model could be applied to the study of the control mechanisms of patients with torso tilt and diagnosis of the change in control mechanisms from patients’ behaviors.

## Introduction

Understanding how humans control their body is essential for effective rehabilitation. Trials have been conducted to elucidate postural control through experiments with humans [1–7] and animals [7–10]. Although experiments have observed the relationships between various factors and resultant behaviors, they have not provided an understanding of associated activity inside the human brain and body. Experiments and simulations complement each other, and simulations would contribute to a better elucidation of the human postural control mechanism. Human postural control to maintain an upright stance is considered to involve both active and passive mechanisms [11–16]. However, passive mechanisms alone are insufficient to maintain posture, given previous studies on ankle stiffness [11–13, 17, 18]. Therefore, we are focusing on developing a neural controller (NC) model to control a human body model.

NC models are designed using two approaches. One approach is system identification [17–22]. The system model is created based on input and output data obtained from experimental results. The model parameters are tuned to minimize the differences between the simulated and experimental results. Researchers have studied the influence on postural control of sensory information [19], muscle stiffness [17], and asymmetries of patients with Parkinson’s disease [20], focusing on ankle joints. The methods were extended for multiple joints, and sources of sensory information [18], muscle stiffness [21], and asymmetries of Parkinson’s patients [22] were studied accordingly. The other approach is forward modeling [23–31]. Unlike system identification, no experimental data are used as input. The developed NC model does not need to simulate experimental data. The performance of the model is evaluated based on whether the features of human movements are reflected. In addition to conventional feed-forward (FF) control in conjunction with feedback (FB) control [23–27], intermittent control has been proposed, in which the controller is intermittently activated [28–30]. It has been reported that FB control by itself can be used to maintain posture [31]. Van der Kooij et al. reported that their controller with FF and FB control could compensate for a neurological time delay of 80 ms [27]. Masani et al. reported that a time delay of 185 ms could be compensated for with only PD control, provided the gain was sufficiently high [31]. We assume that forward modeling, which models postural control without experimental data, is an effective way of understanding the mechanisms of human postural control.

The human body is a very complex system that is driven by hundreds of muscles. Because considering activations of all muscles and skeletal bones has a high calculation cost, torque-driven inverted pendulum models (with 1–3 degrees of freedom (DoF)) have been widely used as models of a human body [18, 19, 21, 23–34]. A simple human body model resembles an inverted pendulum model with 1 DoF for ankle joints. For such a simple model, it is easy to obtain a transfer function from a differential equation [14], which also eliminates the need for large computational resources. The torque around a joint is determined by the forces of the muscles connected to the joint, and activated muscles generate an internal force. Although internal forces influence joint stiffness, which is an important element of postural control, this internal force is excluded when using a torque-driven model. The human control system modulates internal forces; thus, this mechanism should be reflected in an NC model. Therefore, muscle forces should be included. In addition, a human body model should consider the three-dimensional location information of muscles and skeletal bones.

The improved processing speed of computers has enabled simulations of musculoskeletal models in a three-dimensional space [35–43], including simulations that elucidated the mechanisms of stance postural control by forward modeling [35–38]. Clark maintained the standing posture of a musculoskeletal model by using a stretch-reflex controller; however, only forward and backward perturbations were considered [35]. Versteeg et al. proposed a framework for generating the optimal muscle activations of reactive balance [38]. However, they only considered a backward support surface translation as a perturbation. Here, we propose an NC model consisting of FF and FB control [36, 37] following previous studies [23, 25–27]. Because this controller is intended for a musculoskeletal model, FF control (as opposed to torque control) is implemented as the set of muscle activations that can maintain a posture and adjust internal forces. Because the performance of PD control has been confirmed [23, 28–31], FB control is implemented as PD controllers with muscle length and lengthening velocity information. We previously succeeded in maintaining a musculoskeletal model with 70 muscles in an upright posture with a neurological time delay (NTD) of 120 ms [36]. However, only an unperturbed stance was considered in the simulations; the performance of this NC model in response to perturbations was not evaluated.

Because humans in the real world must respond to perturbations, the performance of an NC model must also be evaluated in response to perturbations. Although two types of perturbations (forward and backward) have been considered in previous studies [35, 38], the directions of perturbations that affect humans are not always in the sagittal plane. Therefore, lateral and diagonal perturbations should be considered in addition to forward and backward perturbations. The objective of this study was to investigate the validity of an NC model with FF and FB control in response to multidirectional perturbations. The NC model [36] was used to maintain the stance of a musculoskeletal model. The support surface was translated in multiple directions as perturbations. The performance of the NC model was evaluated based on integrated muscle activations against perturbations and passive ankle stiffness.

## Methods

Simulations were performed using a musculoskeletal model constructed in OpenSim 3.3 (SimTK.org) [41]. The musculoskeletal model was controlled with the NC model [36]. The support surface on which the musculoskeletal model stood was horizontally translated to introduce perturbations. The parameters of the NC model were optimized for each perturbation direction. The integrated muscle activations and passive ankle stiffness during simulations were calculated and used for evaluations.

### Musculoskeletal model

**Fig 1:**
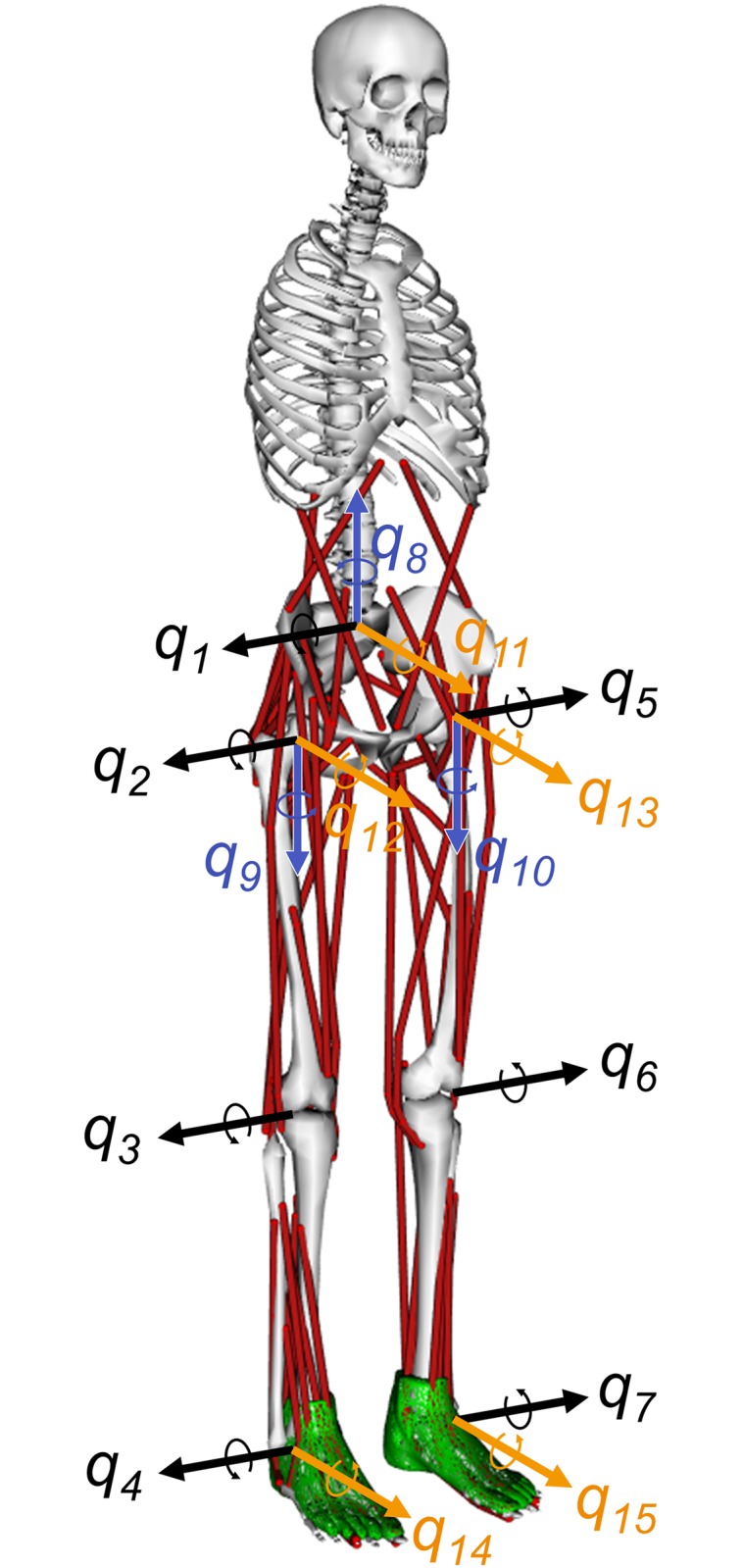
Musculoskeletal model. A musculoskeletal model with 70 muscles and 15 DoF of joints was used. The 35 muscular-tendon actuators were as follows: gluteus medius 1, gluteus medius 2, gluteus medius 3, biceps femoris long head, biceps femoris short head, sartorius, adductor magnus, tensor fasciae latae, pectineus, gracilis, gluteus maximus 1, gluteus maximus 2, gluteus maximus 3, iliacus, psoas major, quadratus femoris, fixme gem, piriformis, rectus femoris, vastus medialis, medial gastrocnemius, lateral gastrocnemius, soleus, tibialis posterior, flexor digitorum longus, flexor hallucius longus, tibialis anterior, peroneus brevis, peroneus longus, peroneus tertius, extensor digitorum longus, extensor hallucius longus, erector spinae, internal oblique, and external oblique. The gluteus medius muscle and the gluteus maximus muscle were each composed of three muscular-tendon actuators. The musculoskeletal model had the following movements: trunk bending (*q*_1_), trunk leaning to side (*q*_11_), trunk twisting (*q*_8_), hip flexion (*q*_2_), hip adduction and abduction (*q*_12_ and *q*_13_), hip rotation (*q*_9_ and *q*_10_), knee flexion (*q*_3_ and *q*_6_), ankle flexion (*q*_4_ and *q*_7_), and ankle inversion and eversion (*q*_14_ and *q*_15_).

A standing musculoskeletal model was influenced by perturbations in the anterior-posterior, lateral, and diagonal directions. Eight DoF of joints were added to a musculoskeletal model used in our previous study [36], and a musculoskeletal model was developed with 70 muscular-tendon actuators [44] and 15 DoF of joints (Fig 1). The parameters of body segments (e.g., mass and moments of inertia), muscles (e.g., location and maximum isometric force), and joint designs were derived from a model proposed by Delp et al. [45]. The model has been widely used for simulation of gait [42, 46], landing [43], and perturbed stance [35, 38–40]. The contact between a foot and the ground was modeled as the contact between a three-dimensional mesh and a plane. The three-dimensional mesh of a foot was derived from a cadaver foot [47]. The floor reaction force was calculated using an elastic foundation force model [48]. The contact parameters were derived from a previous study by DeMers et al. [43]. Refer to [45, 47] for details of kinematics and [44, 45, 48] for details of dynamics.

### Neural controller

The NC model consists of FF control for muscle activations to adopt a posture and FB control to compensate for the differences between the target posture and the actual posture [36]. FB control is indispensable because it is known to play a vital role in postural control [49]. In addition, previous studies have indicated the possible existence of FF control [19, 50], and Fitzpatrick et al. reported that FB control alone is not sufficient for posture stabilization in response to perturbations [19]. Focusing on this knowledge, we designed the NC model to include both FF control and FB control. The NC model maintains the standing posture of a musculoskeletal model with a neurological time delay (NTD) of 120 ms, the value of which was chosen based on previous studies [31, 51–53]. Simulations without perturbations were performed in our previous study [36]. However, the NC model considers proprioceptive information, which is a primary sensory FB resource in normal conditions [19, 54]. Therefore, the NC model is likely applicable for a perturbed stance.

**Fig 2:**
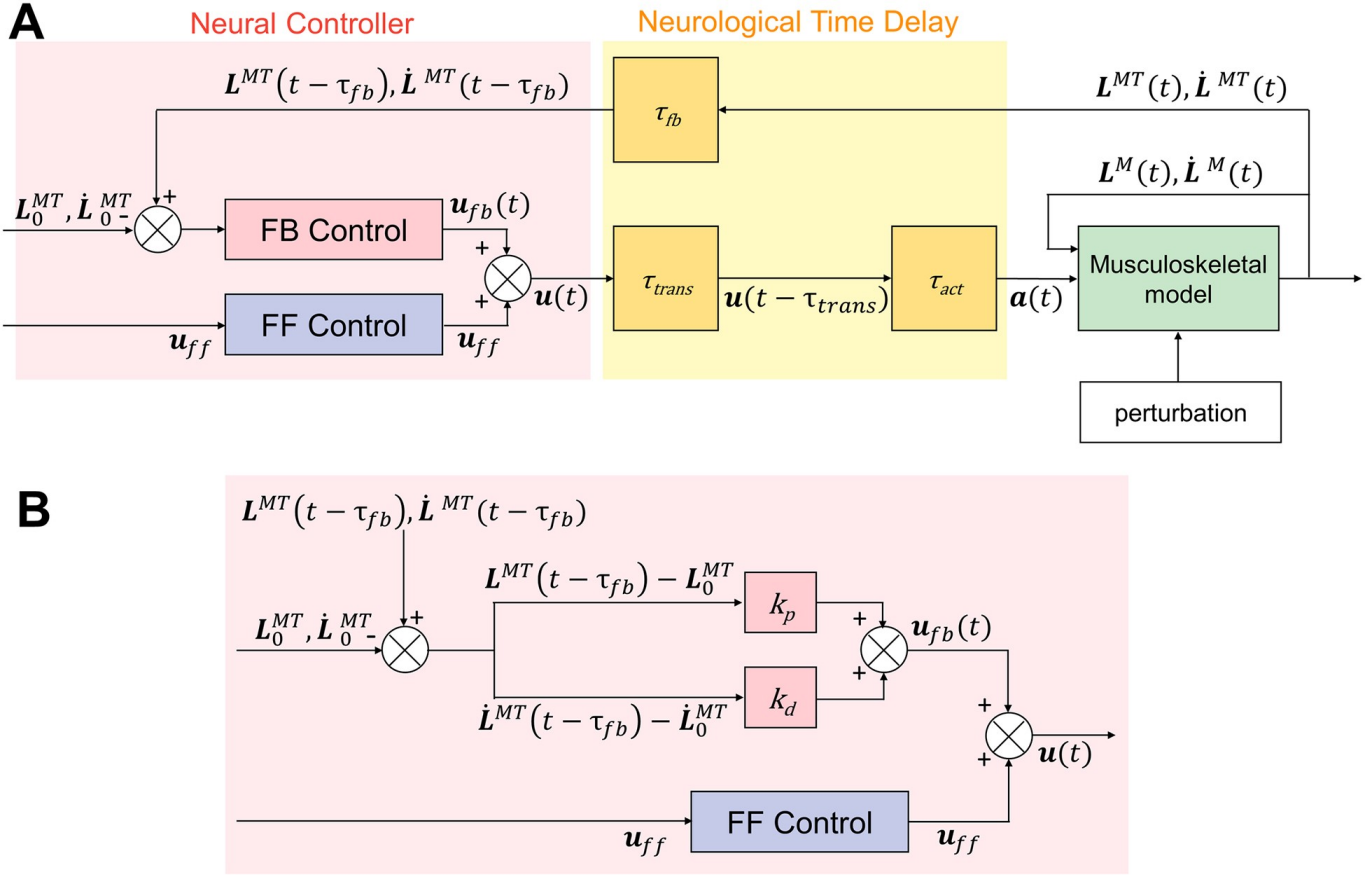
Diagram of a neural controller. (A) This NC model, proposed in a previous study [36], consists of FF and FB control. ***u***, ***u***_*fb*_, and ***u***_*ff*_ are the total, FB, and FF controls, respectively. ***a*** denotes muscle activation. ***L***^*MT*^ and ${\dot{L}}^{MT}$ are the length and lengthening velocity of the muscular-tendon actuators, respectively. $L_{0}^{MT}$ and ${\dot{L}}_{0}^{MT}$ are the initial values of the length and lengthening velocity of the muscular-tendon actuators (${\dot{L}}_{0}^{MT}={\ddot{L}}_{0}^{MT}=0$), respectively. ***L***^*M*^ and ${\dot{L}}^{M}$ are the length and lengthening velocity of muscle fibers, respectively. (B) FB control is implemented as PD controllers using proprioceptive information (muscle length and lengthening velocity). ***k***_*p*_ and ***k***_*d*_ are PD gains.

The NC diagram is illustrated in Fig 2. The initial muscle lengths and lengthening velocities, muscle lengths and velocities at time *t* (time delay of *τ*_*fb*_) and FF control components are used as inputs to the NC model. The initial muscle lengths and lengthening velocities are target values determined using the initial posture of the musculoskeletal model at *t* = 0. The output is the total control of muscle activations ***u***(*t*), which is the sum of FF control components ***u***_*ff*_ and FB control components at time *t* ***u***_*fb*_(*t*) (Eq (1)).

$$\begin{array}{c} \begin{array}{c} u\left(t\right)=u_{ff}+u_{fb}\left(t\right) \end{array} \end{array}$$ (1)

#### Feed-forward control

FF control is used for muscle activations to enable a stance. An FF control component of the *i*th muscle *u*_*ff, i*_ is kept constant during the simulation (Eq (2)).

$$u_{ff,i}=c_{i}$$ (2)

*c*_*i*_ is a constant value. When ∥***u***_*ff*_∥^2^ ($∥u_{ff}{∥}^{2}=c_{1}^{2}+c_{2}^{2}+...+c_{i}^{2}$) is large, the stiffness of the body is high.

#### Feedback control

It is impossible to maintain a musculoskeletal model in a standing posture using only FF control. FB control is used to compensate for differences between the target posture and the actual posture. Information regarding the current posture is available from muscle spindles, which can detect changes in muscle length and muscle lengthening velocity. This response represents PD control information, the performance of which has been tested in previous studies [27–31]. Therefore, FB controllers are implemented as PD controllers that use muscle lengths and lengthening velocities as FB information. The FB control component of the *i*th muscle at time *t* *u*_*fb*,*i*_(*t*) is the sum of a component of proportional control and a component of differential control using muscle length (Eq (3)).

$$\begin{array}{c} \begin{array}{cc} u_{fb,i}\left(t\right) & =k_{p,i}\frac{\left(L_{i}^{MT}\left(t-\tau_{fb}\right)-L_{i,0}^{MT}\right)}{L_{i,0}^{MT}}+k_{d,i}\frac{\left({\dot{L}}_{i}^{MT}\left(t-\tau_{fb}\right)-{\dot{L}}_{i,0}^{MT}\right)}{V_{i,max}} \end{array} \end{array}$$ (3)

$L_{i}^{MT}\left(t\right)$ is the length of the *i*th muscular-tendon actuator at time *t*, and ${\dot{L}}_{i}^{MT}\left(t\right)$ is the lengthening velocity of the *i*th muscular-tendon actuator at time *t*. *V*_*i*,*max*_ is the maximum limit of the lengthening velocity of the *i*th muscle. *V*_*i*,*max*_ is the parameter of a muscular-tendon actuator that is preset in the musculoskeletal model. *k*_*p*,*i*_ and *k*_*d*,*i*_ are the PD gains for FB control.

#### Neurological time delay

We adopted a maximum NTD of 120 ms. This NTD included an FB delay *τ*_*fb*_, a transmission delay *τ*_*trans*_, and an activation dynamics delay *τ*_*act*_. *τ*_*fb*_ is the delay associated with the sensory receptors’ receipt of the sensory information. *τ*_*trans*_ is the delay associated with the NC’s transmission of control information to the neurons that control muscle activation. *τ*_*act*_ is the delay between the muscles receiving control signals and generating force. *τ*_*fb*_ and *τ*_*trans*_ are constant time delays, which were set to 40 ms in accordance with a previous study by Masani et al. [31]. *τ*_*act*_ is a variable time delay, which depends on muscle activity. The activation dynamics of muscles were modeled by a first-order differential equation (Eqs (4) and (5)) [51]. The activation and deactivation time constants were set to 10 and 40 ms, respectively [52, 53].

$$\begin{array}{c} \begin{array}{c} {\dot{a}}_{i}\left(t\right)=\frac{u_{i}\left(t-\tau_{trans}\right)-a_{i}\left(t\right)}{\tau (a_{i}\left(t\right),u_{i}\left(t-\tau_{trans}\right))} \end{array} \end{array}$$ (4)

$$\begin{array}{c} \tau \left(a_{i}\left(t\right),u_{i}\left(t-\tau_{trans}\right)\right)=\{\begin{array}{cc} t_{act}\left(0.5+1.5a_{i}\left(t\right)\right) & \left(u_{i}\left(t-\tau_{trans}\right)>a_{i}\left(t\right)\right)\\ t_{deact}/\left(0.5+1.5a_{i}\left(t\right)\right) & \left(u_{i}\left(t-\tau_{trans}\right)\leq a_{i}\left(t\right)\right) \end{array} \end{array}$$ (5)

*u*_*i*_ is the output from the NC model for the *i*th muscle, and *a*_*i*_ is the muscle activation of the *i*th muscle. *t*_*act*_ and *t*_*deact*_ were set to 10 and 40 ms, respectively.

### Forward dynamics simulations

Support surface translations have been used in experimental studies [2, 5, 7, 55–60] and are easy to reproduce in simulations. In some studies, multidirectional perturbations have been implemented as support surface translations in 12 directions [55–60].

**Fig 3:**
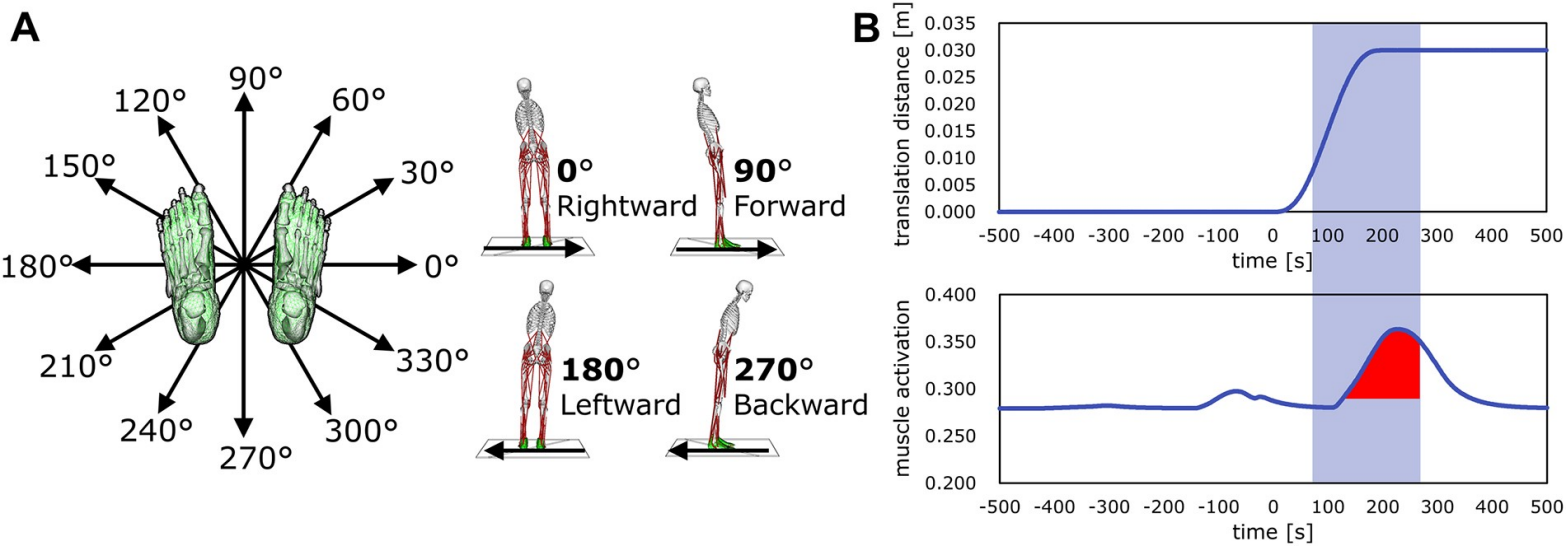
Horizontal support surface translations as perturbations and index of magnitude of muscle responses against perturbations. (A) Perturbations were applied in the form of horizontal support surface translations in 12 directions separated by 30°. A rightward translation was defined as 0°after the definition of Henry et al. [55]. When a 0°translation was applied, the surface moved in a rightward direction, and the body tilted leftward. (B) The perturbation was implemented with an s-shaped step function. The support surface was translated 3 cm in 200 ms. The velocity and acceleration at *t* = 0 ms and 200 ms were 0. Muscle activations from 70–270 ms were observed to evaluate simulated muscle responses (indicated in red).

In this study, the support surface on which the musculoskeletal model stood was horizontally translated to introduce perturbations. The support surface was translated in 12 directions separated by 30°(Fig 3). The magnitude of the translations was 3 cm in 200 ms. The translational distance of 3 cm was smaller than that used in experimental studies [2, 5, 7, 55–60]. However, translational distances used in musculoskeletal simulations [35, 38–40] tend to be smaller than those employed in experimental studies [2, 5, 7, 55–60]. In these simulation studies, the actual features of humans have not been completely reproduced (e.g., models have used a rigid foot without metatarsophalangeal joints and a torso without arms and spine joints). The limitations of the reproduction limit the magnitudes of perturbations. However, it is important to use appropriate conditions for models rather than identical conditions in model and human studies. We selected a translational distance of 3 cm in 200 ms because the peak velocity and acceleration of the translation were the same as those in prior musculoskeletal simulations [35, 38–40]; the current study additionally considered multidirectional perturbations.

To implement the perturbation, an s-shaped step function prepared in OpenSim 3.3 was used. This function was modified to translate the support surface 3 cm in 200 ms. The translation distance *y*(*t*) can be written as Eq (6).

$$\begin{array}{c} \begin{array}{c} y\left(t\right)=30{\left(t/200\right)}^{3}-45{\left(t/200\right)}^{4}+18{\left(t/200\right)}^{5}\;\left(0\leq t\leq 200\right) \end{array} \end{array}$$ (6)

*y*(*t*) changes smoothly from 0 to 3 when *t* changes from 0 to 200. This function has first and second derivatives *y*′(0) = *y*′(200) = 0 and *y*″(0) = *y*″(200) = 0, respectively. The shape of the translation function is indicated in Fig 3.

### Parameter adjustment

The number of unknown parameters to be adjusted was 210 (70-dimensional ***u***_*ff*_, 70-dimensional ***k***_*p*_, and 70-dimensional ***k***_*d*_). Even muscles with similar attachments required different muscle activations to maintain a certain posture [61]. Therefore, 35 muscle activations for 35 types of muscles were adjusted individually for ***u***_*ff*_. Because muscles are not symmetrically attached around joints, PD gains for flexion and extension were separately considered (although lumbar and hip joints were ball joints, only their flexion and extension were considered, similar to knee and ankle joints). Muscles formed 11 groups: lumbar extensor, lumbar flexor, hip extensor, hip flexor, knee extensor, knee flexor, ankle extensor, ankle flexor, subtalar evertor, subtalar invertor, and biarticular. Muscles in the same muscle group had identical PD gains. To address the increase in the DoF of joints, more muscle groups were used in the current model than the 7 groups used in our previous study [36]. The number of parameters adjusted was 57 (35 for FF control and 22 for PD gains of FB control).

Determining a suitable solution for all parameters was challenging because of a large search range and because the NTD was larger than 100 ms. Therefore, parameter adjustment was carried out in two stages. Because ***u***_*ff*_ is constant and independent of NTD, only ***u***_*ff*_ was calculated in the first stage. PD gains were optimized for each ***u***_*ff*_ in the second stage.

**Fig 4:**
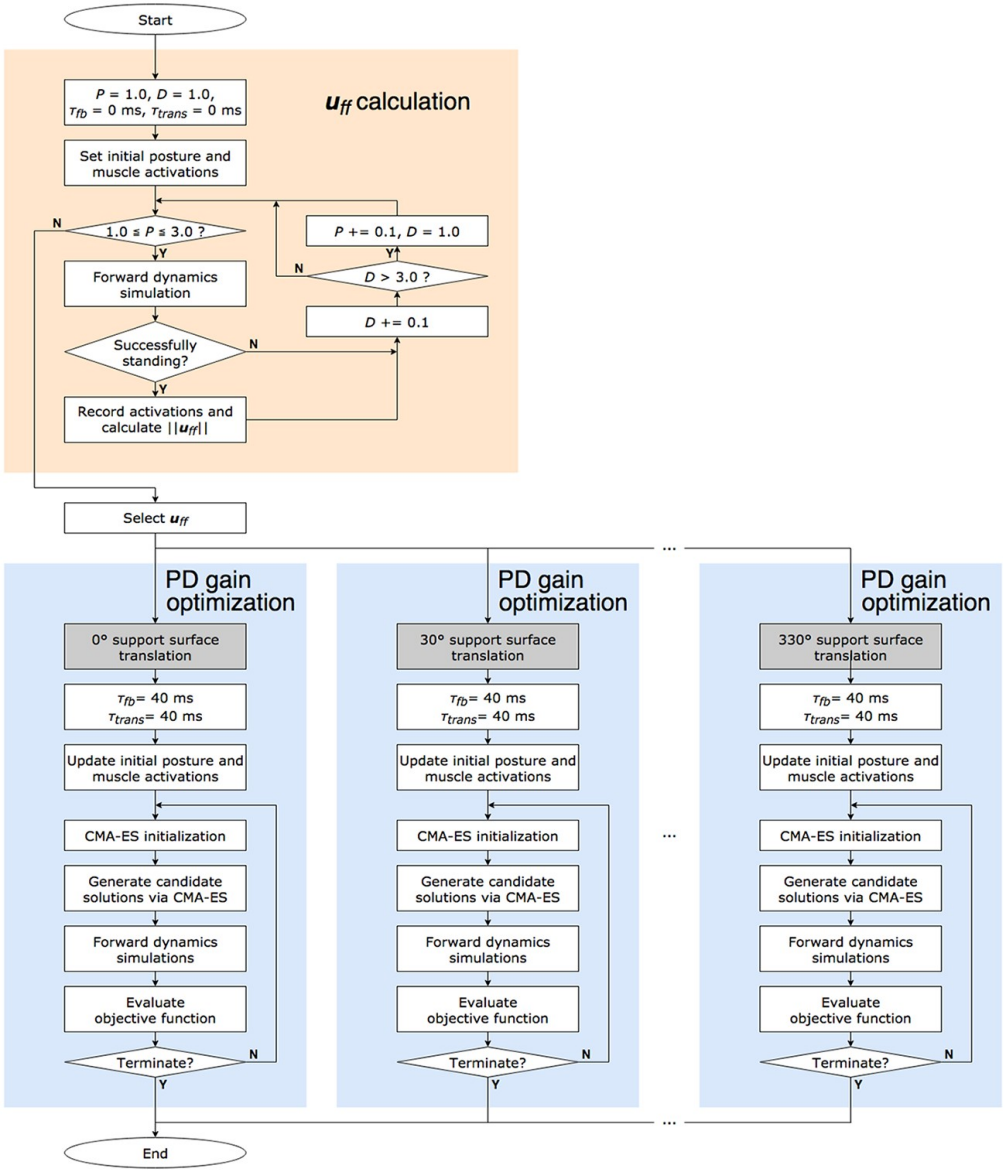
Parameter adjustment algorithm. ***u***_*ff*_ candidates were calculated (indicated in orange). From results of simulations with 0-ms *τ*_*fb*_ and *τ*_*trans*_, ***u***_*ff*_ candidates were obtained. After selecting a ***u***_*ff*_, optimizations were performed for each direction of perturbations with CMA-ES (indicated in blue). Note that only one ***u***_*ff*_ is shown in this figure. A total of 12 different optimizations were performed for a different ***u***_*ff*_.

In an experimental study [55] used for comparison with the simulation results, subjects were translated in 1 of 12 directions randomly in the horizontal plane (we used these 12 directions, as described in the “Evaluation index” section of the current paper). We considered that subjects could not adopt appropriate muscle activations and joint angles before perturbations. Therefore, ***u***_*ff*_ was not optimized for the perturbation directions. Humans can determine perturbation direction from sensory information, including that available from the sole of the foot. This information reaches the brain at approximately the same time as the muscle-length information and is available to improve postural control. Therefore, we assumed that humans can use direction-specific feedback control, which was empirically tuned. Optimizations to adjust PD gains were performed for each ***u***_*ff*_ and for each perturbation direction (Fig 4). Note that these methods of parameter adjustment had a high calculation cost, but real-time performance was not required in this study.

#### *u*_*ff*_ calculation

***u***_*ff*_ was constant and independent of NTD. We developed a musculoskeletal model with a standing posture with a small NTD. When the musculoskeletal model maintained a stance, a ***u***_*ff*_ candidate was determined based on the muscle activations during the simulation.

***u***_*ff*_ = **0** and some PD gains were set in the NC model, and a simulation was performed with 0-ms *τ*_*fb*_ and *τ*_*trans*_. When a musculoskeletal model stood for 5000 ms, the value of muscle activations was integrated, and a ***u***_*ff*_ candidate was generated (Eq (7)).

$$\begin{array}{c} u_{ff,i}=c_{i}=\frac{\int_{t_{1}}^{t_{2}}a_{i}\left(t\right)dt}{t_{2}-t_{1}} \end{array}$$ (7)

*u*_*ff*,*i*_ is the FF control component of the *i*th muscle and is constant (*c*_*i*_). *t*_1_ and *t*_2_ determine the range of muscle activations for the ***u***_*ff*_ calculation and were set to 5000 and 3000 ms, respectively, based on [36]. *a*_*i*_(*t*) is the muscle activation of the *i*th muscle at time *t*.

The PD gains of the NC model were set as shown in Eq (8).

$$\left[k_{p,i},k_{d,i}\right]=\{\begin{array}{l} {}[0.50P,0.23D],\;\text{group}=\text{lumbar}\;\text{extensor}\\ {}[0.48P,0.11D],\;\text{group}=\text{lumbar}\;\text{flexor}\\ {}[0.45P,0.05D],\;\text{group}=\text{hip}\;\text{extensor}\\ {}[0.50P,0.16D],\;\text{group}=\text{hip}\;\text{flexor}\\ {}[0.33P,0.05D],\;\text{group}=\text{knee}\;\text{extensor}\\ {}[0.28P,0.23D],\;\text{group}=\text{knee}\;\text{flexor}\\ {}[0.17P,0.06D],\;\text{group}=\text{ankle}\;\text{extensor}\\ {}[0.30P,0.27D],\;\text{group}=\text{ankle}\;\text{flexor}\\ {}[0.50P,0.11D],\;\text{group}=\text{subtalar}\;\text{evertor}\\ {}[0.49P,0.05D],\;\text{group}=\text{subtalar}\;\text{invertor}\\ {}[0.39P,0.05D],\;\text{group}=\text{biarticular} \end{array}$$ (8)

The ratio was obtained through optimization of an unperturbed stance simulation (to minimize the objective function *J* (Eq (9)) with *τ*_*fb*_ = 0, *τ*_*trans*_ = 0, and ***u***_*ff*_ = **0**). Note that the ratio was not used in the following “PD gain optimization” section. We varied *P* and *D* of Eq (8) within the range of 1.0–3.0 at increments of 0.1. The time range was defined by *t*_1_ and *t*_2_, and the increments were the same as those in our previous study [36]. The range of *P* and *D* (1.0–3.0) was selected to obtain greater ***u***_*ff*_ compared with that when the same range was used in our previous study [36]. Note that an infinite number of ***u***_*ff*_ candidates can be obtained by changing the range of *P* and *D*.

In our previous study, which focused on an unperturbed stance [36], nine ***u***_*ff*_ values were selected at equal intervals of ∥***u***_*ff*_∥^2^ and used for simulations. In the results, ***u***_*ff*_ of ∥***u***_*ff*_∥^2^ = 2.07 provided a stance most similar to that of humans. Therefore, in the current study, ***u***_*ff*_ were also selected at equal intervals of ∥***u***_*ff*_∥^2^, including ***u***_*ff*_, such that ∥***u***_*ff*_∥^2^ was close to 2.07.

#### PD gain optimization

Some values of ***u***_*ff*_ were selected from the ***u***_*ff*_ candidates. PD gains were optimized for an optimal stance for each ***u***_*ff*_ and direction. Covariance matrix adaptation evolution strategy (CMA-ES) [62] was used for optimization to find PD gains that minimized the objective function *J*. CMA-ES is an evolutionary algorithm for solving nonlinear black-box optimization problems that has been applied to optimize parameters of a controller for gait generation [46]. The population size λ and initial standard deviation *σ* were set to 18 and 0.005, respectively, for fast convergence. A maximal iteration number of 1500 was defined. Furthermore, the simulation evaluated 18 candidate solutions generated by CMA-ES in parallel with each iteration.

$$\begin{array}{c} J=w_{fail}J_{fail}+w_{pos}J_{pos} \end{array}$$ (9)

$$\begin{array}{c} J_{fail}=\frac{1}{T_{fall}}\left(T_{simu}-T_{fall}\right) \end{array}$$ (10)

$$\begin{array}{c} T_{fall}=\{\begin{array}{cc} T_{stop} & \left(h_{CoM}<0.9\;m\right)\\ T_{simu} & \left(h_{CoM}\geq 0.9\;m\right) \end{array} \end{array}$$ (11)

$$\begin{array}{c} J_{pos}=\sum_{j=1}^{15}\int_{0}^{T_{fall}}\left(\theta_{j}\left(t\right)-\theta_{j}\left(0\right)\right)dt \end{array}$$ (12)

The objective function *J* is the weighted sum of *J*_*fail*_ for evaluating the time for which the musculoskeletal model can stand, and *J*_*pos*_ is used for evaluating the pose of a musculoskeletal model. *w*_*fail*_ and *w*_*pos*_ are the weights of the two evaluation axes and were set as 10,000 and 1, respectively. *T*_*simu*_ is the simulation time and was set as 5000 ms, based on [36]. *T*_*fall*_ is the time when the height of the CoM (*h*_*CoM*_) is less than 0.9 m. When the musculoskeletal model bows (i.e., folds forward) with 60°hip flexion, the height of the CoM is 0.92 m. Because the perturbations were expected not to cause such large tilt angles, the threshold of the CoM height was set as 0.9 m. If *h*_*CoM*_ is constantly greater than or equal to 0.9 m (if a musculoskeletal model can maintain a stance posture for *T*_*simu*_), *T*_*fall*_ is equal to *T*_*simu*_, and *J*_*fail*_ = 0. *J*_*pos*_ is the sum of the time-integrated deviations of joint angles. *θ*_*j*_(*t*) is the angle of *j*th joint at time *t*. For information on *θ*_*j*_(0), see S1 File.

### Evaluation index

Henry et al. asked healthy subjects to stand on a movable surface that was translated in 12 directions separated by 30°, with a magnitude of 9 cm in 200 ms [55]. The 12 different perturbation directions were randomly presented. The electromyographic (EMG) responses of the subjects were measured, and the magnitudes of muscle responses in response to perturbations were calculated. To confirm whether the magnitudes of responses in the current study were biologically plausible, the simulation results obtained in our study were compared with the experimental results obtained by Henry et al. The same evaluation index as that used in their study was calculated from the muscle activations in our simulations.

Activations of six left-sided muscles were observed: erector spinae (ESP), rectus femoris (RFM), tensor fasciae latae (TFL), tibialis anterior (TIB), soleus (SOL), and medial gastrocnemius (MGS).

An example of perturbation and reactive muscle activations is shown in Fig 3. The evaluation index was calculated from muscle activations 70–270 ms after the onset of perturbations. The integrated value was calculated with Eq (13).

$$\begin{array}{c} \begin{array}{c} integrated\;muscle\;activation=\int_{0}^{270}\left(a_{i}\left(t\right)-a_{baseline,i}\right)dt \end{array} \end{array}$$ (13)

*a*_*i*_(*t*) is the muscle activation of the *i*th muscle at time *t*, and *a*_*baseline*,*i*_ is the mean value of *a*_*i*_(*t*) 50–150 ms before the onset of perturbations. Similar ranges were observed in several previous studies [55–57]. The differences between the muscle activation values and baseline were time-integrated in the range of 200 ms as the magnitudes of muscle responses against perturbations. For each muscle, the integrated values were normalized between zero and one by defining the maximum value among all 12 directions as one. Then, the normalized values were plotted.

To confirm whether the magnitudes of the simulated muscle responses were consistent with human experimental results, cosine similarity was employed. When the simulation results and experimental results were similar, the cosine similarity value was high. The evaluation index calculated with Eq (14) for 12 directions can be written as a 12-dimensional vector. The evaluation index for 12 directions was normalized between -1 and +1 as a general normalization for cosine similarity. The cosine similarity between a 12-dimensional vector of the simulation results ***v***_*sim*_ and that of human experimental results ***v***_*exp*_ was calculated using Eq (14).

$$\begin{array}{c} similarity=\frac{v_{sim}\cdot v_{exp}}{|v_{sim}∥v_{exp}|} \end{array}$$ (14)

***v***_*sim*_ is a 12-dimensional vector obtained by normalizing 12 evaluation indexes from simulated muscle activations in our simulations. ***v***_*exp*_ is a 12-dimensional vector obtained by normalizing 12 evaluation indexes from EMGs measured in human experiments in a previous study [55]. The cosine similarity varies between -1 and +1. When two vectors are identical, the cosine similarity value is +1.

To evaluate the cosine similarity between ***v***_*sim*_ and ***v***_*exp*_, cosine similarity values were calculated between 100,000 vectors with random values ***v***_*rand*_ and ***v***_*exp*_.

$$\begin{array}{c} similarity=\frac{v_{rand}\cdot v_{exp}}{|v_{rand}∥v_{exp}|} \end{array}$$ (15)

***v***_*sim*_ is a 12-dimensional vector consisting of random values between -1 and 1. A cumulative distribution function was calculated with the mean and the standard deviations of the 100,000 trials, with the assumption that the distribution was normal. A cumulative distribution of cosine similarity values between a 12-dimensional vector of simulation results and that of experimental results was calculated and used to assess whether the cosine similarity was high.

Passive joint stiffness has been measured, including passive ankle stiffness, and it has been reported that passive stiffness alone cannot stabilize an upright posture [11–13, 17, 18]. To confirm whether the simulated passive ankle stiffness was biologically plausible, the passive ankle stiffness obtained in our simulations and in a previous study were compared. We observed passive ankle stiffness when the support surface was translated backward (270°, the most used direction in prior studies). The observed time range was 0–70 ms, which denotes the time from the onset of perturbation to the onset of the observation of muscle activations.

In previous studies [11–13], the relative stiffness calculated with Eq (16) was less than one.

$$\begin{array}{c} Relative\;stiffness=\frac{2K}{mgh} \end{array}$$ (16)

*K* is the passive ankle stiffness. *m* is the mass of the musculoskeletal model (above an ankle joint), *g* is the gravitational acceleration, and *h* is the deviation between the height of the CoM and that of an ankle joint. *mgh* is referred to as the critical stiffness, which is the minimum stiffness required to maintain a standing posture without changes in muscle activation.

To calculate the relative stiffness, the passive ankle stiffness *K* was calculated from the relationship between the ankle angle *θ*_*ankle*_ and the passive ankle torque *T*_*passive*_. The muscle forces of the musculoskeletal model were calculated using Eq (17).

$$\begin{array}{c} F_{i}=f_{o,i}^{M}\left(a_{i}f^{L}\left({\tilde{l}}_{i}^{M}\right)f^{V}\left({\tilde{v}}_{i}^{M}\right)+f^{\text{PE}}\left({\tilde{l}}_{i}^{M}\right)\right) \end{array}$$ (17)

$f_{o}^{M}$ is the maximum isometric force, *a* denotes activation, ${\text{f}}^{L}\left({\tilde{l}}^{M}\right)$ is the active-force-length curve, ${\text{f}}^{V}\left({\tilde{v}}^{M}\right)$ is the force-velocity curve, and ${\text{f}}^{\text{PE}}\left({\tilde{l}}^{M}\right)$ is the passive-force-length curve. $\tilde{l}$ and $\tilde{v}$ represent the normalized muscle length and lengthening velocity. *i* is the number of muscles. The details of the curves are described in [44].

The passive ankle torque *T*_*passive*_ was affected by two components of Eq (17). One was the passive force (${\text{f}}^{\text{PE}}\left({\tilde{l}}_{i}^{M}\right)$). When the length of a muscle was greater than the optimal length, a passive force was generated. The other component was an active force derived from ***u***_*ff*_ ($a_{i}{\text{f}}^{L}\left({\tilde{l}}_{i}^{M}\right){\text{f}}^{V}\left({\tilde{v}}_{i}^{M}\right)$). Even for the same activation, the active force derived from ***u***_*ff*_ could be changed depending on $\tilde{l}$ and $\tilde{v}$. Therefore, the passive ankle torque *T*_*passive*_ was calculated with Eq (18).

$$\begin{array}{c} T_{passive}=\sum_{i}f_{o,i}^{M}\left(a_{ff,i}{\text{f}}^{L}\left({\tilde{l}}_{i}^{M}\right){\text{f}}^{V}\left({\tilde{v}}_{i}^{M}\right)+{\text{f}}^{\text{PE}}\left({\tilde{l}}_{i}^{M}\right)\right)\times \left(ma_{i}\right) \end{array}$$ (18)

*a*_*ff, i*_ is the activation derived from ***u***_*ff*_, and *ma*_*i*_ is the moment arm.

The passive ankle torque *T*_*passive*_ was modeled with Eq (19), as used in a previous study [11], to calculate parameters such as the passive ankle stiffness *K*.

$$\begin{array}{c} T_{passive}=K\theta_{ankle}+B{\dot{\theta }}_{ankle}+I{\ddot{\theta }}_{ankle}+C \end{array}$$ (19)

*K*, *B*, *I*, and *C* denote the stiffness, viscosity, moment of inertia, and a constant value, respectively. Note that a constant value *C* was added to the equation used in the previous study [11] because *θ*_*ankle*_ at *t* = 0 ms was not always the same. Linear least squares regression was used to estimate *K*, *B*, *I*, and *C* from *T*_*passive*_ and *θ*_*ankle*_.

## Results

### Results of parameter adjustment

Our simulations, conducted with only FB control, under the condition of *τ*_*fb*_ = 0, *τ*_*trans*_ = 0, and ***u***_*ff*_ = **0** generated 316 ***u***_*ff*_ candidates. Seven ***u***_*ff*_ values were selected at equal intervals of ∥***u***_*ff*_∥^2^ (∥***u***_*ff*_∥^2^ = 1.00, 2.01, 2.98, 4.02, 4.97, 5.95, and 6.39), following our previous study [36] (S2 File). Because the ***u***_*ff*_ calculation method failed to yield ***u***_*ff*_, which satisfied ∥***u***_*ff*_∥^2^ > 6.39 owing to the changes in the numbers of DoF of joints and muscle groups, the maximum ∥***u***_*ff*_∥^2^ was 6.39 in this study.

**Fig 5:**
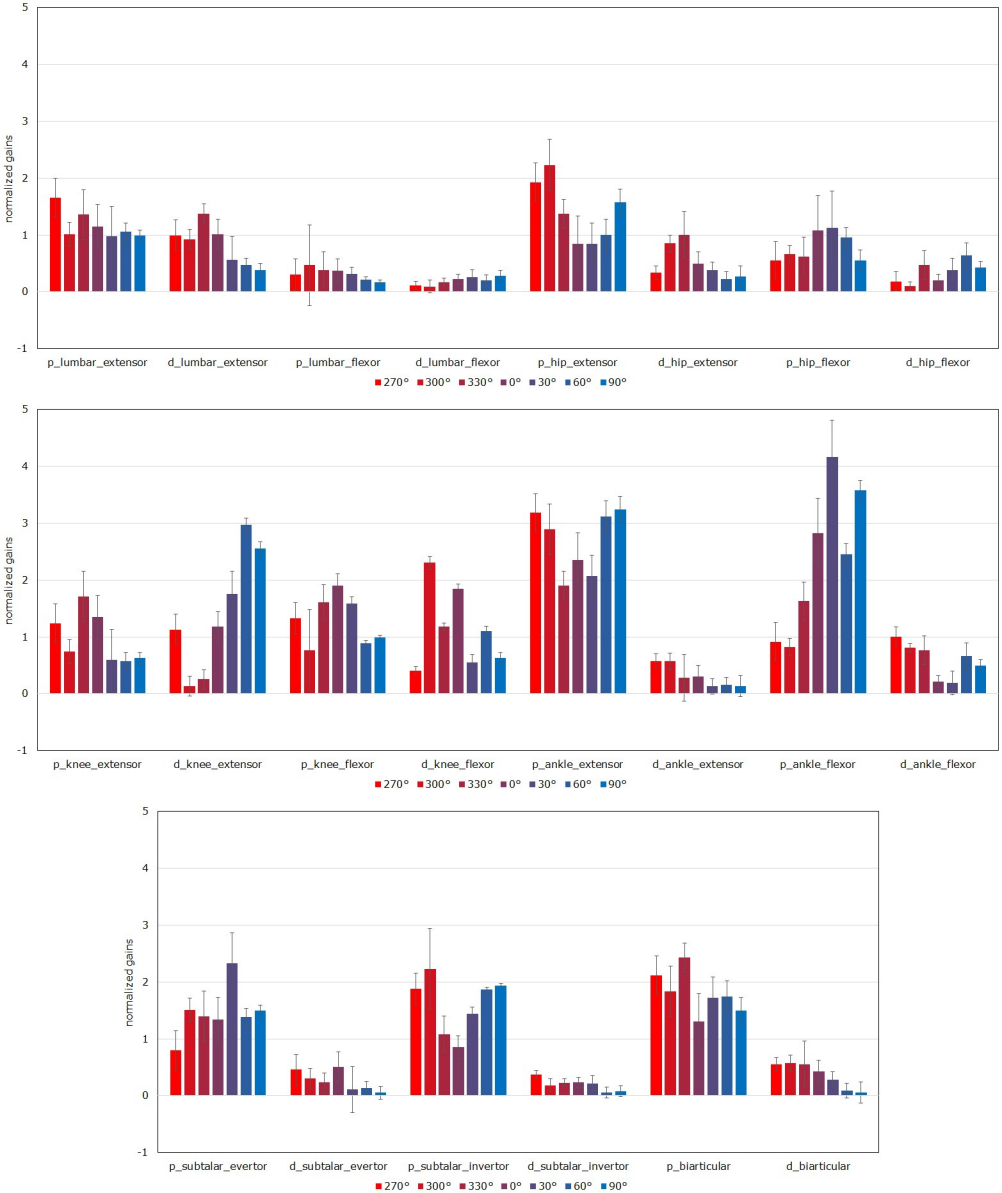
Trends of PD gains for directions. The values for the graph were calculated as follows. A total of 22 PD gain values for each ***u***_*ff*_ and perturbation direction were divided by the mean of 22 values (normalization). The mean and standard deviations of the normalized values for ***u***_*ff*_ were calculated and plotted in the graph. “p_lumbar_extensor” is the P gain for the lumbar extensor group, and “d_lumbar_extensor” is the D gain for the lumbar extensor group. Perturbation directions 120°–240°are omitted because of the symmetry of simulations.

After performing 84 optimizations for 7 ***u***_*ff*_ and 12 directions of perturbations (Fig 4), the PD gains for which the musculoskeletal model could maintain a stance in all conditions, except for ∥***u***_*ff*_∥^2^ = 1.00, were successfully obtained (S1 Video, S3 File). The trends of the obtained PD gains were calculated and plotted in Fig 5.

### Results of evaluation

#### Magnitudes of muscle responses against perturbations

**Fig 6:**
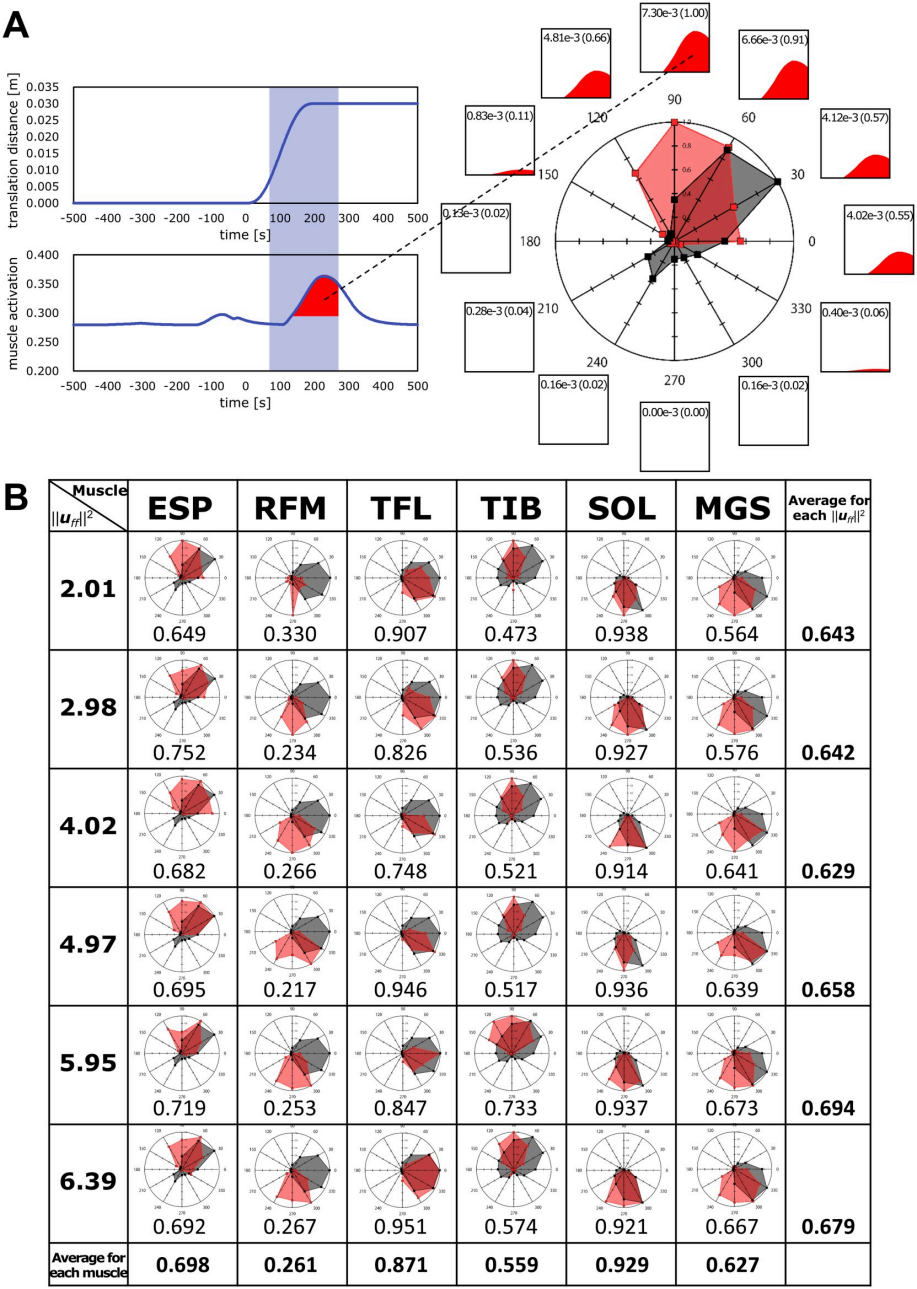
Magnitudes of muscle responses against perturbations. (A) Muscle activations in the range of 70–270 ms after the onset of perturbations were integrated and used as the index of the magnitudes of muscle activations. This time range was the same as that in a human experimental study [55]. When ∥***u***_*ff*_∥^2^ = 2.01, the ESP muscle was activated for a forward translation (90°). The value of integrated muscle activation calculated with Eq (13) was 7.30e-3 s. As 7.30e-3 s was the largest integrated value for the 12 directions, we normalized the integrated values to values of 0 to 1, such that 7.30e-3 s was defined as 1. The 12 boxes around the radar chart indicate integrated ESP muscle activations against perturbations in each direction. The number in the box denotes the value of integrated ESP muscle activation, and the number within parentheses is the normalized value. In the radar chart, the red shaded area denotes the simulation results, and the black shaded area denotes the human experimental results [55]. (B) Each row of the table contains radar charts for each ∥***u***_*ff*_∥^2^. Each column contains radar charts for each muscle (see the “Evaluation index” section for muscle names). The numbers below each radar chart are the cosine similarity values for each condition. The average of the cosine similarity for each ∥***u***_*ff*_∥^2^ and for each muscle are indicated in the right side and bottom.

The magnitudes of muscle responses against perturbations are shown in Fig 6. The radar charts indicate how left-sided muscles responded to perturbations. Fig 6 shows 36 radar charts for six observed muscles and six values of ***u***_*ff*_.

As an example, we explain the upper left radar chart that shows how left-sided ESP responded to perturbations when ***u***_*ff*_ = 2.01. In our simulations, ESP was maximally activated when a 90°support surface translation occurred. In contrast, ESP was not activated in response to backward translations. In the experiments, ESP was maximally activated when a 30°support surface translation occurred. ESP was not activated for backward and leftward translations. Note that the simulated and human experimental responses were independently normalized to the maximum response among the 12 directions. The cosine similarity value for the 12-dimensional vectors of the simulation and human experimental results was 0.649.

The mean of the cosine similarity values for all ***u***_*ff*_ is listed in Table 1. We also indicate the mean and standard deviations of the cosine similarity values by using the vectors of the human experimental results and vectors with random values and a cumulative distribution. Note that we assumed the distribution to be normal.

**Table 1:** Cosine similarity values.

	**ESP**	**RFM**	**TFL**
*Simulation*	0.698	0.261	0.871
*Random*	1.40e-3±0.288	1.70e-3±0.289	-2.91e-4±0.290
*Cumulative distribution*	0.992	0.815	0.999
	**TIB**	**SOL**	**MGS**
*Simulation*	0.559	0.929	0.627
*Random*	1.31e-5±0.289	1.64e-4±0.290	-3.94e-4±0.289
*Cumulative* *distribution*	0.973	0.999	0.985

Simulation: the mean of the cosine similarity values obtained using the vectors of the simulation results and human experimental results for all ***u***_*ff*_. Random: the mean and standard deviations of the cosine similarity values obtained using the vectors of experimental results and random values. Cumulative distribution: the probability that the mean of the cosine similarity values obtained using the vectors of simulation results and experimental results is larger than or equal to the mean of the cosine similarity value with a vector of the experimental results and a vector with random values.

#### Passive ankle stiffness

All calculated parameters (stiffness, viscosity, moment of inertia, and constant value) and relative stiffness are indicated in Table 2. Ankle stiffness increased with an increase in ∥***u***_*ff*_∥^2^, except for ∥***u***_*ff*_∥^2^ = 4.97 and ∥***u***_*ff*_∥^2^ = 5.95. All relative stiffness values were smaller than one.

**Table 2:** Passive response parameters and relative stiffness.

	Stiffness	Viscosity	Inertia	Constant	Relative Stiffness
∥***u***_*ff*_∥^2^ = 2.01	1.17	0.0508	2.17e-3	-9.64	0.208
∥***u***_*ff*_∥^2^ = 2.98	1.42	0.0921	-1.34e-3	2.10	0.252
∥***u***_*ff*_∥^2^ = 4.02	2.40	0.146	1.75e-4	-1.06	0.425
∥***u***_*ff*_∥^2^ = 4.97	3.73	0.121	-2.49e-4	-5.25	0.662
∥***u***_*ff*_∥^2^ = 5.95	3.55	0.142	-4.85e-4	-4.74	0.631
∥***u***_*ff*_∥^2^ = 6.39	4.55	0.134	-7.86e-4	-9.45	0.808

The response to a backward support surface translation (500–570 ms) was modeled with Eq (19). The calculated stiffness *K* (Nm/deg), viscosity *B* (Nms/deg), moment of inertia *I* (Nms^2^/deg), and constant value *C* are indicated in this table. The relative stiffness was calculated with Eq (16). The mass of the musculoskeletal model (mass above an ankle joint) *m* was 72.0 kg, and the gravitational acceleration *g* was 9.80665 m/s^2^. The deviation between the height of the CoM and that of an ankle joint *h* was 0.914 m (the average of *h* for all simulations was 0.914±3.65e-4 m).

## Discussion

### Relationships between PD gains and perturbation directions

When a muscle is lengthened by perturbations, the muscle has to be activated and generate a force to maintain posture. In this study, the P gain for a muscle lengthened by perturbations was expected to be large and that for an antagonist was expected to be small.

When the surface was translated backward, the feet were also translated backward with the surface while the upper half of the body remained in its initial position. This condition caused a forward lean of the posture due to flexion of the knee and ankle, as observed in a previous study [63]. Therefore, we expected the P gains for the knee extensor group and ankle flexor group to be large in response to a backward translation. The simulation results were consistent with this expectation (Fig 5). The P gains for the knee flexors for 30°–90°were not smaller than those for 270°–330°. We suggest that this result is due to the structural features of the knee joint, which is almost fully extended when a human adopts an upright posture. Except for the P gains for the ankle extensor group (30°–90°), the relationship between the P gains and perturbation directions appears consistent with anatomical features.

Because the same PD gains were assigned to right-sided and left-sided muscles in this study, we expected no directionality of the P gains for each subtalar group. However, when observing the P gains from 270°to 90°in Fig 5, the subtalar evertor group showed an inverted U-shape, whereas the subtalar invertor group showed a U-shape. The gains for the subtalar groups were considered to be optimized as gains for antagonists.

Because the musculoskeletal model was a 3D multilink system, the relationships between the P gains and perturbation directions were expected to weaken with increasing distance between the muscles and the support surface. The relationships between the P gains for the lumbar groups (abdominal/back muscles) and perturbation directions were weak. Because the graph for the hip extensor group was U-shaped and that for the hip flexor group had an inverted U-shape, the P gains for the hip groups were considered to be optimized as gains for antagonists.

The biarticular muscle group consisted of biarticular muscles of several body parts. Therefore, it was difficult to evaluate the gains for the biarticular group.

We hypothesize that the P gains were appropriately optimized for each perturbation direction. Currently, assessing the validity of the directional features of D gains is challenging because no other study of a musculoskeletal model has considered multidirectional perturbations. With greater ***u***_*ff*_, the directional features of the PD gains would be exhibited more profoundly.

### Directional features of magnitudes of muscle responses

The cumulative distributions of ESP, RFM, TFL, TIB, SOL, and MGS were larger than 0.97 (Table 1). That is, the cosine similarity values obtained using simulated results and human experimental results were larger than those obtained using random values and experimental results, with a probability of 0.97 or higher. Therefore, we infer that the magnitudes of the muscle responses were consistent with human experimental results [55].

The cosine similarity value and the cumulative distribution of RFM was 0.261 and 0.815, respectively; these values were the smallest among the 6 muscles. This result occurred because the maximally activated direction of the simulated results (270°–300°, backward) and the experimental results (0°, rightward) were orthogonal. Considering the anatomical orientation of RFM, it appears to be maximally activated in response to a backward support-surface translation, which causes the greatest muscle lengthening. However, a study of humans reported that RFM is maximally active orthogonal to the direction of greatest lengthening, and this observation was assumed to be due to complex control mechanisms that involve the interaction of peripheral and central processes [55]. We suggest that the absence of complex control mechanisms in the NC model was reflected in the differences between the RFM responses in the simulations and those in the human experiments. In contrast, the mean cosine similarity values for each ∥***u***_*ff*_∥^2^ varied from 0.629 (∥***u***_*ff*_∥^2^ = 4.02) to 0.694 (∥***u***_*ff*_∥^2^ = 5.95). No clear relationship was observed between the size of ∥***u***_*ff*_∥^2^ and the cosine similarity values. Thus, even if the body stiffness changes, the trends of the muscle responses remain unchanged.

### Compensation for neurological time delay and perturbations by feed-forward control

In this study, we obtained PD gains that maintained the stance of a musculoskeletal model for all conditions (except for ∥***u***_*ff*_∥^2^ = 1.00). Only in the conditions with ∥***u***_*ff*_∥^2^ = 1.00, the lowest value of ∥***u***_*ff*_∥^2^, did the NC model fail to make the musculoskeletal model stand. The results suggest that the NC model can make the musculoskeletal model maintain a posture if ∥***u***_*ff*_∥^2^ is sufficiently large; that is, a certain degree of stiffness compensates for NTD and perturbations and enables the maintenance of a posture. This finding is consistent with the results of our previous study [36].

In our previous study, ∥***u***_*ff*_∥^2^ = 0.89 enabled a musculoskeletal model to stand. In contrast, in this study, ∥***u***_*ff*_∥^2^ = 1.00 (>0.89) could not make the model stand. We suggest that perturbations and the increase in the DoF of joints made the musculoskeletal model more unstable; therefore, a higher stiffness was required.

### Challenges in finding parameters to maintain a musculoskeletal model in a standing posture

In our previous study [36], we performed simulations of an unperturbed stance of a musculoskeletal model with 7 DoF of joints. In this study, we performed simulations of a perturbed stance of a musculoskeletal model with 15 DoF of joints. We assumed that the conditions for parameters (***u***_*ff*_, ***k***_*p*_, and ***k***_*d*_) were stricter because of the perturbations and the increase in DoF of joints.

For example, consider a condition in which part of the DoF of joints is missing. When hip adduction/abduction and rotation are locked, the muscles for hip adduction/abduction or rotation (e.g., adductor longus) have a slight influence on the maintenance of a stance, regardless of the degree of muscle outputs; that is, when the DoF of joints is low, tuning the PD gains for some muscles is unnecessary.

The NC model had to not only make a musculoskeletal model maintain a standing posture but also compensate for perturbations. In particular, for a forward support-surface translation, the projection of the CoM was likely to be beyond the base of support because of the structure of the feet.

Therefore, the conditions for parameters were stricter than those of our previous study [36]. However, we succeeded in determining the parameters required to make a musculoskeletal model maintain a standing posture by using the framework of the NC model, which validates the effectiveness of the NC model.

### Joint stiffness change caused by *u*_*ff*_ change

Calculated ankle stiffness increased with an increase in ∥***u***_*ff*_∥^2^ (except for ∥***u***_*ff*_∥^2^ = 4.97 and ∥***u***_*ff*_∥^2^ = 5.95); that is, FF control in the NC model can adjust joint stiffness (Table 2). Joint stiffness has been measured in previous studies, which demonstrated that passive ankle stiffness alone is insufficient for maintaining a standing posture [11–13]. The obtained relative stiffness in the current study were smaller than one (0.208–0.808), which is consistent with experimental results.

The calculated moment of inertia (*I*) was positive (∥***u***_*ff*_∥^2^ = 2.01 and 4.02) or negative (∥***u***_*ff*_∥^2^ = 2.98, 4.97, 5.95 and 6.39). We calculated the passive ankle torque using Eq (18). The muscle force was affected by the muscle length and lengthening velocity, but it was not affected by acceleration. We consider that the estimated *I* was close to zero because *T*_*passive*_ could be fitted only with *Kθ*_*ankle*_, $B{\dot{\theta }}_{ankle}$, and *C*.

## Conclusions

The objective of this study was to investigate the validity of an NC model with FF and FB control in response to multidirectional perturbations. We developed a standing musculoskeletal model with the NC model and translated the support surface as perturbations. We determined parameters that maintained the stance of the musculoskeletal model for each perturbation direction. Although the parameter conditions were stricter than the unperturbed stance simulations [36], we succeeded in determining parameters that maintained a stance in response to perturbations for six ***u***_*ff*_. The trends in the magnitudes of muscle responses in simulations were consistent with those of human experimental results [55], and the relative stiffness for all conditions was smaller than one, supporting the validity of the NC model.

The direction of maximal RFM activity in simulations was orthogonal to that in experiments. We suggest that this orthogonality was caused by the RFM responses not being based on simple FB control, as described in a human experimental study [55]. To elucidate the responses further, it would be necessary to consider functions and features of the human body as a multilink system, including prediction and learning.

We considered only proprioceptive information (muscle length and lengthening velocity) as a major resource for FB control. Visual, vestibular, and other sensory information was not implemented. However, it was suggested that the weights for sensory FB information change when a perturbation occurs [33]. Simulations with several sources of sensory FB information would help clarify how each type of sensory FB contributes to compensation for perturbations and how the contribution changes in response changes in perturbations.

This study indicates that musculoskeletal simulations are useful for understanding the underlying mechanisms of human postural control, especially for asymmetrical motions. We anticipate that we can model the impairment of specific patient populations by adjusting the parameters of an NC model and a musculoskeletal model based on patients’ behaviors. However, improvements in models and methods of parameter adjustment are required to efficiently simulate such populations.

## Supporting information

S1 Video Perturbed stance simulation video. (MP4)

S1 File Initial posture. The information of the initial pose of pelvis (pelvis_tilt, pelvis_list, pelvis_rotation, pelvis_tx, pelvis_ty, and pelvis_tz) and initial joint angles (hip_flexion_r (*q*_2_), hip_adduction_r (*q*_12_), hip_rotation_r (*q*_9_), knee_angle_r (*q*_3_), ankle_angle_r (*q*_4_), subtalar_angle_r (*q*_14_), hip_flexion_l (*q*_5_), hip_adduction_l (*q*_13_), hip_rotation_l (*q*_10_), knee_angle_l (*q*_6_), ankle_angle_l (*q*_7_), subtalar_angle_l (*q*_15_), lumbar_extension (*q*_1_), lumbar_bending (*q*_11_), and lumbar_rotation (*q*_8_)) are indicated.
(CSV)

S2 File Selected *u*_*ff*_. A total of 70 types of muscle activations used as ***u***_*ff*_ are indicated.
(CSV)

S3 File Optimized PD gains. P and D gains for 11 muscle groups are indicated. For example, p_lumbar_extension is the P gain for the lumber extension muscle group, and d_lumbar_extension is the D gain for the lumbar extension muscle group. Because the musculoskeletal model is symmetrical, the directions of 120°–240°are omitted (e.g., left-sided muscle activations against a 120°translation are the same as those against a 60°translation).
(CSV)
